# Modulation of Inflammatory Response by Electromagnetic Field Stimulation in Traumatic Brain Injury in Yucatan Swine

**DOI:** 10.26502/jsr.10020338

**Published:** 2024-01-31

**Authors:** Yssel Mendoza-Mari, Vikrant Rai, Mohamed M. Radwan, James Brazdzionis, David A Connett, Dan E Miulli, Devendra K Agrawal

**Affiliations:** Department of Translational Research, College of Osteopathic Medicine of the Pacific, Western University of Health Sciences, Pomona CA 91766

**Keywords:** Electromagnetic field, Immunomodulation, Inflammation, NLRP3 inflammasome, Swine model, Traumatic Brain Injury

## Abstract

Traumatic brain injury is a leading cause of disability and death worldwide and represents a high economic burden for families and national health systems. After mechanical impact to the head, the first stage of the damage comprising edema, physical damage, and cell loss gives rise to a second phase characterized by glial activation, increased oxidative stress and excitotoxicity, mitochondrial damage, and exacerbated neuroinflammatory state, among other molecular calamities. Inflammation strongly influences the molecular events involved in the pathogenesis of TBI. Therefore, several components of the inflammatory cascade have been targeted in experimental therapies. Application of Electromagnetic Field (EMF) stimulation has been found to be effective in some inflammatory conditions. However, its effect in the neuronal recovery after TBI is not known. In this pilot study, Yucatan miniswine were subjected to TBI using controlled cortical impact approach. EMF stimulation via a helmet was applied immediately or two days after mechanical impact. Three weeks later, inflammatory markers were assessed in the brain tissues of injured and contralateral non-injured areas of control and EMF-treated animals by histomorphometry, immunohistochemistry, RT-qPCR, Western blot, and ELISA. Our results revealed that EMF stimulation induced beneficial effect with the preservation of neuronal tissue morphology as well as the reduction of inflammatory markers at the transcriptional and translational levels. Immediate EMF application showed better resolution of inflammation. Although further studies are warranted, our findings contribute to the notion that EMF stimulation could be an effective therapeutic approach in TBI patients.

## Introduction

Traumatic Brain Injury (TBI) induces a set of physical, molecular, cognitive, and behavioral disorders that arise from an external mechanical impact on the head. This condition is considered one of the leading causes of long-term disability and mortality worldwide, a high economic burden for families and national health systems, and a risk factor for the development of other pathologies in the central nervous system (CNS), including Alzheimer’s disease [1]. According to CDC reports, in the United States, there were approximately 214,110 TBI-related hospitalizations in the year 2020 and 69,473 TBI-related deaths in 2021 [2].

TBI constitutes a two-stages event where the initial phase occurs instantly after injury and comprises physical damage to the brain, edema, cell loss, extra and intraparenchymal hemorrhages, and focal or diffuse axonal trauma [3]. The second phase is the most extended stage, and it could last from minutes to months or years [4]. From a biochemical point of view, it is characterized by an increase in reactive oxygen species (ROS) and other oxidative stress markers, mitochondrial dysfunction, altered neuronal metabolism, ion imbalance, membrane disruption, cytoskeleton damage, DNA fragmentation, and disrupted neural transmission [5]. This second phase of damage amplifies the deleterious effects of the initial mechanical trauma and is ultimately responsible for progressive neurodegeneration and long-term disorders that reduce the patient's quality of life.

Neuroinflammation plays a leading role following TBI. Astrocytes, and especially microglia, are cellular mediators of this inflammatory response after brain trauma [6]. During the first moments, activation of microglia induces the release of downstream pro-inflammatory cytokines, providing immunological protection against invading pathogens and deleterious internal molecules [7]. When this process becomes dysregulated, the initial neuroprotective effect switches to an exacerbated inflammatory response that contributes to neurological symptoms and neurodegeneration [5].

NLR family pyrin domain containing 3 (NLRP3) inflammasome is actively involved in the neuroinflammatory response after TBI [8]. This multiprotein complex comprises three protein subunits: NLRP3 as the sensor molecule, ASC as the adaptor protein, and caspase-1 (CASP1) as effector protein [9]. The assembly of the structure allows the self-cleavage and subsequent activation of pro-caspase-1 into CASP1 and this enzyme in turn activates interleukin-1 β (IL-1β) and interleukin-18 (IL-18) by proteolytical cleavage of the pro-peptides [10]. These pro-inflammatory cytokines are then released into the neuronal milieu, exacerbating the inflammatory environment [11] and promoting cell pyroptosis [12].

To this date, several therapeutic agents including corticosteroids, excitatory amino acids inhibitors, calcium channel blockers, free radical scavengers, etc. have been used to regulate the molecular damages of TBI [13-15], but unfortunately, none have proven to be effective in human trials. Among non-pharmacological approaches, Electromagnetic Field (EMF) application has proven to provide neuroprotection in terms of modulating ROS in an animal model of ischemic stroke [16,17]. At the molecular level, EMF also showed a beneficial impact regarding nitrous oxide modulation [18], apoptosis [19], superoxide production [20], microcirculation [21] inflammation [22], and apoptosis [23]. Despite these encouraging results, reports regarding EMF effects in TBI are still limited. Recently, our group developed an animal model of TBI in pigs, based on a Controlled Cortical Impact (CCI), in which EMF was applied, demonstrating that the evaluation of brain neuronal circuits can be appropriately assessed with this technology [24-28].

In this study, we evaluated the impact of EMF stimulation on inflammatory cascade, specifically on the molecular components of the NLRP3 complex and other related factors, in swine subjected to TBI. Although with limitations, our results demonstrated that EMF could modulate the expression of molecules involved in this pathological pathway, contributing to reducing the levels of proinflammatory cytokines and therefore attenuating the deleterious effects in the brain of the EMF-stimulated swine.

## Materials and Methods

### Animal model:

**M**ale Yucatan minipigs (Premier BioSource, Ramona, CA) were used, according to guidelines of the National Institutes of Health and USDA for the care and use of experimental animals. The protocol for this study was approved by the Western University of Health Science Institutional Animal Care and Use Committee under protocol number R23IACUC003. Animals were maintained on a normal diet with unrestricted access to water. TBI model and EMF application were done as described [26-28]. Three animals were extemporaneously used in this pilot study and all of them were subjected to mechanical impact to develop TBI. Animal 1 did not receive EMF therapeutical stimulations; therefore, it is considered an injured untreated control. Animal 2 received the EMF therapy two days after TBI induction (delayed approach) and animal 3 received the EMF 20 minutes following TBI induction (immediate approach). Electromagnetic therapeutic stimulation and signal detection were performed as previously described [26-28].

### Tissue harvest:

Blood samples were obtained pre-operatively and post-operatively on the day of surgery, at different time points throughout the study, and on the sacrifice day for quantifying circulating markers related to TBI pathology. Brain cortex samples from the impacted area (injury site, IS) and contralateral control area (non-injury site, NO IS) were collected and preserved in 10% formalin (6764254, ThermoFisher Scientific, Waltham, Massachusetts, USA) for histological analysis, in RNAlater (AM7021, ThermoFisher Scientific, Waltham, Massachusetts, USA) for total RNA extraction, and at −80°C for protein isolation.

### Histology processing and staining:

After 24 h in formalin, tissue fragments were immersed in consecutive solutions of ethanol, xylene, and paraffin wax in a tissue processor Tissue-Tek VII (Sakura Finetek, Torrance, CA, USA). Paraffin blocks were generated and 5 μm sections were obtained using a Leica RM2265 rotary microtome (Leica^™^, Wetzlar, Germany) placed on the glass slide and then incubated at 60°C for one hour. Before staining and immunohistochemistry, paraffin-embedded tissues were deparaffinized and hydrated according to standard protocols in our laboratory. For H&E staining, slides were incubated in hematoxylin for 90 sec and in eosin for 3 minutes. The stained sections were mounted with a xylene-based mounting medium Cytoseal (23-244257, ThermoFisher Scientific, Waltham, MA, USA). Slides were scanned using a Leica DM6 light microscope (Leica^™^, Wetzlar, Germany) with a scale of 100 μm. At least three adjacent sections from each tissue and 3-5 fields per section were scanned. All the scanned sections were analyzed by two independent observers to detect signs of inflammation, apoptosis, pyknotic nuclei, vacuolation, etc.

### Quantitative Real-Time Polymerase Chain Reaction (RT-qPCR):

Approximately 50 mg of brain tissue from each sample was used to isolate total RNA using TRIZOL (T9424, Millipore Sigma, Burlington, MA, USA) following the manufacturer’s instruction protocol in our laboratory. RNA pellet was resuspended in 30 μL of nuclease-free water (BP561-1, ThermoFisher Scientific, Waltham, MA, USA) and RNA yield was quantified using Nanodrop 2000 Spectrophotometer (Thermo Fisher, Waltham, MA, USA). Two micrograms of total RNA were used to synthesize complementary DNA (cDNA) using AzuraQuant^™^ cDNA Synthesis Kit (AZ-1996, Azura Genomics Inc., Raynham, MA, USA) according to manufacturer’s instruction using a T100^™^ Thermal Cycler (Bio-Rad Laboratories, Hercules, CA, USA). The cDNAs were diluted 1:20 in nuclease-free water and qPCR reactions were prepared in a final volume of 10 μL and in triplicate using AzuraView^™^ GreenFast qPCR Blue Mix LR (AZ-2350, Azura Genomics Inc., Raynham, MA, USA). Amplification was carried out in a C1000^™^ Thermal Cycler (Bio-Rad Laboratories, Hercules, CA, USA) and the cycling conditions were the following: 3 minutes at 95°C for initial denaturation, 40 cycles of 10 sec at 95°C (denaturation), 30 sec at 60°C (annealing/extension) followed by melting curve analysis. The primers for genes of interest and housekeeping gene (Table 1) were purchased from Integrated DNA Technologies (Coralville, IA, USA). After normalization with 18S, relative gene expression was calculated using 2^−ΔΔCT^ method.

### Immunohistochemistry (IHC):

After slides were deparaffinized and rehydrated, antigen retrieval was performed by heating the samples in 1% citrate buffer (C9999-1000ML, Millipore Sigma, Burlington, MA, USA) in a commercial steamer for 45 minutes. The slides were cooled for another 45 minutes and washed with 1x phosphate-buffered saline (PBS) (BP39920, ThermoFisher Scientific, Waltham, MA, USA) for 5 minutes. Endogenous peroxidase activity was blocked by incubating slides with 3% hydrogen peroxide (H1009, Millipore Sigma, Burlington, MA, USA) for 15 minutes at room temperature. After washing two times in 1x PBS of 5 minutes each, tissue sections were treated for 1 h at room temperature with ready-to-use blocking solutions: Normal Horse Serum for mouse antibodies (S-2000-20), Normal Goat Serum for rabbit antibodies (S-1000-20) and Normal Rabbit Serum for goat antibody (S-5000-20) (Vector Laboratories, Newark, CA USA) and were subsequently incubated with primary antibodies (Table 2) overnight at 4°C. The day after, samples were rinsed twice in 1x PBS for 5 minutes and were incubated for 1 h at room temperature with ready-to-use biotinylated secondary antibodies (Table 2). Tissue sections were washed two times with 1x PBS for 5 minutes each, followed by incubation with VECTASTAIN^®^ ABC-HRP Kit (PK-4000, Vector Laboratories, Newark, CA USA) for 30 minutes at room temperature. After rinsing with 1x PBS solution as previously described, tissue sections were incubated with AEC Substrate Kit, Peroxidase (HRP), (3-amino-9-ethylcarbazole) (SK-4200, Vector Laboratories, Newark, CA USA) for 10 minutes until color development and the reaction was stopped by immersing slides in tap water. Counterstaining with hematoxylin was done for approximately 1 minute and the sections were mounted using ADVANTAGE Mounting Media (NB300A, Innovex Biosciences, Pinole, CA, USA). At least three images from each tissue section were manually analyzed using Fiji Image J Software (version 1.54J, NIH, USA) [29] to semi-quantify the mean intensity and percentage of stained area [30].

### Western blot

Approximately 100 mg of brain tissue from each sample was used to isolate total proteins. Tissue fragments were disrupted in 1 mL of 1x PBS solution supplemented with a protease inhibitor cocktail (Pierce Protease Inhibitor Mini Tablets, A32953, ThermoFisher Scientific, Waltham, MA, USA) using a tissue disruptor (PowerGen 125, ThermoFisher Scientific, Waltham, MA, USA). After complete dissociation, samples were centrifuged at 4°C for 10 minutes to remove insoluble fragments. Supernatants were transferred to new Eppendorf tubes and total protein concentrations were estimated by the Bradford method [31] using Bio-Rad Protein Assay Kit II (5000002, Bio-Rad Laboratories, Hercules, CA, USA). Twenty μg of total protein were loaded and run on SDS gel (4–15% Mini-PROTEAN TGX Precast Protein Gels (4561084, Bio-Rad Laboratories, Hercules, CA, USA) and then transferred to PVDF membrane (1620177, Bio-Rad Laboratories, Hercules, CA, USA) according to standard procedure. The appropriate transfer was checked by Ponceau Red staining (P7170, Millipore Sigma, Burlington, MA, USA) and afterward, the membranes were blocked for 1 h at room temperature in the blocking solution in 1x Tris Buffered Saline (TBS) (50-489-119, ThermoFisher Scientific, Waltham, MA, USA) supplemented with 0.1% Tween20 (P1379, Millipore Sigma, Burlington, MA, USA) and 5% skimmed milk (1706404, Bio-Rad Laboratories, Hercules, CA, USA). Membranes were incubated with primary antibodies (Table 2) prepared in the blocking solution overnight at 4°C with gentle agitation. After that, membranes were rinsed three times for 5 minutes in washing solution consisting of 1xTBS / 0.1% Tween 20, followed by incubation with appropriate secondary antibodies (Table 2) for 1 h at room temperature with gentle agitation. Then, membranes were washed, and signals were developed with Pierce ECL Western Blotting Substrate (32106, ThermoFisher Scientific Waltham, MA, USA). Images were obtained in a ChemiDoc XRS+ System (Bio-Rad Laboratories, Hercules, CA, USA) and processed using Fiji Image J Software (version 1.54J, NIH, USA) [29]. β-actin was used as a house-keeping protein to ensure that the same amount of protein was applied for all samples.

### ELISA quantification

Biochemical markers for TBI were quantified in serum samples collected before surgery and at different time points during the study and in brain cortex samples collected during the final surgery. Homogenates from IS and NO IS tissues were obtained as previously described. Commercial kits for Neuron-Specific Enolase (NSE) (MBS040255, MyBioSource, San Diego, CA, USA), Glial Fibrillary Acidic Protein (GFAP) (LS-F22386, LSBio Shirley, MA, USA), Ubiquitin Carboxy-terminal Hydrolase (UCHL1) (LS-F12898-1, LSBio Shirley, MA, USA), Myelin Basic Protein (MBP) (LS-F22414, LSBio Shirley, MA, USA), IL-1β (MBS2021728, MyBioSource, San Diego, CA, USA), IL-6 (MBS765708, MyBioSource, San Diego, CA, USA), TNF-α (MBS161499, San Diego, CA, USA) and Total Antioxidant Capacity (TAC) (ab65329, Abcam, Waltham, MA, USA) were used according to manufacturer’s instructions. For tissue-derived samples, results are expressed as a concentration of marker per mg of total protein.

### Statistical Analysis

Data were analyzed using GraphPad Prism 10 for Windows (version 10.1.1) and are represented as mean ± standard deviation. The normality of data was verified by Shapiro Wilk’s test. For each swine, comparisons between injured (IS) *vs* non-injured (NO IS) sites were performed using Student’s t-test. Comparisons among IS from all swine were carried out using One-way ANOVA and Tukey’s posthoc test. For all analyses a p-value < 0.05 was accepted as statistically significant. For two groups comparisons, differences were represented by *p <0.05, **p<0.01, ***p <0.001 and ****p <0.0001. For three groups comparisons, different letters indicate significant differences for at least p < 0.05.

## Results

### Histological analysis

**Figure 1:**
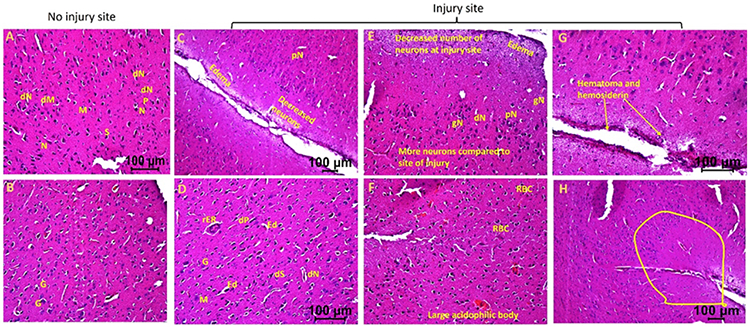
Hematoxylin and eosin staining in swine with TBI with no EMF application (swine 1). dN- Deformed Neuron, dS- Deformed Stellate cell, dM- deformed Microglia, dP- Deformed Pyramidal cells, pN- Neurons with pyknosis, gN- Ghost Neuron, Ed- Edema around neuron and stellate cells, rER- Regenerating Endoplasmic Reticulum, M- Microglia, G- Granular cell. These are represented histological pictures of all images scanned in this swine. The images were scanned at 100μm.

H&E staining of the brain tissues from the injury site of swine not treated with EMF (hereafter swine 1) showed an increased population of deformed neurons, stellate cells, microglia, and pyramidal cells, neurons with pyknosis, ghost neurons, edema around neurons and stellate cells, and regenerating endoplasmic reticulum compared to the tissues from contralateral hemisphere with no injury. Further, injury site tissues showed an increased presence of foci of inflammation. Furthermore, there was increased edema at the site of injury and around neurons and stellate cells, fewer normal appearing neurons, presence of hematoma, immune cells, and hemosiderin at the site of injury. The injured tissues also revealed an increased number of granular cells, and large acidophilic mass surrounding dark apoptotic nuclei (Figure 1).

**Figure 2:**
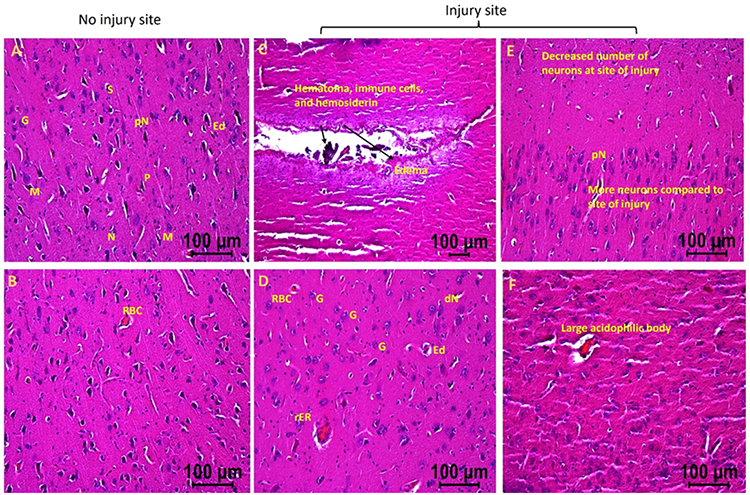
H&E staining in swine with TBI with EMF application after 2 days (swine 2). dN- Deformed Neuron, dS- Deformed Stellate cell, dM- deformed Microglia, dP- Deformed Pyramidal cells, pN- Neurons with pyknosis, gN- Ghost Neuron, Ed- Edema around neuron and stellate cells, rER- Regenerating Endoplasmic Reticulum, M- Microglia, G- Granular cell. These are represented images of all images scanned in this swine. The images were scanned at 100μm.

In the swine where EMF was applied after 2 days of injury (hereafter swine 2), the injured site tissues revealed hematoma, hemosiderin deposition, RBCs, increased number of immune cells, decreased number of normal appearing neurons at the site of injury, pyknotic neurons, degenerating neuron, large acidophilic bodies, edema around the injury site and neurons, and regenerating neurons compared to contralateral tissue without injury (Figure 2). These pathological findings were less severe in swine 2 compared to swine 1. Swine 2 also revealed thrombosis and dividing microglia at the site of injury compared to the non-injured site of the same swine. The non-injured tissues of swine 2 showed many normal appearing pyramidal cells, stellate cells, microglia, and granular cells with a few abnormal cells, normal appearing neurons with a few with pyknosis, minimal edema around the cells, and minimal evidence of RBCs and immune cells.

The injured tissues from swine with TBI and EMF applied just after injury (hereafter swine 3) revealed minimal to no edema, the presence of many near normal neurons, microglia, pyramidal cells, and granular cells with a few with deformed morphology. The edema around neuron and stellate cells decreased in some areas while more in other areas (Figures 3a and 3b). The injured tissues also revealed the presence of dividing microglia, thrombosis, immune cells, and hemosiderin deposition but were lesser than the injured tissues of swine 1 and 2. The number of neurons with pyknosis, large acidophilic bodies surrounding dark apoptotic nuclei, and the number of deformed granular cells decreased compared to the tissues from swine 1 and swine 2 (Figures 3a and 3b). The non-injured tissues from swine 3 showed normal appearing pyramidal cells, stellate cells, microglia, and granular cells with a few abnormal cells. Overall, the injured site tissues’ pathological findings improved with EMF application and were better when EMF was applied just after injury compared to EMF applied 2 days after injury. The non-injured tissues revealed better histology in swine 2 and swine 3 compared to swine 1 and in swine 3 compared to swine 2.

### RT-qPCR

**Figure 4:**
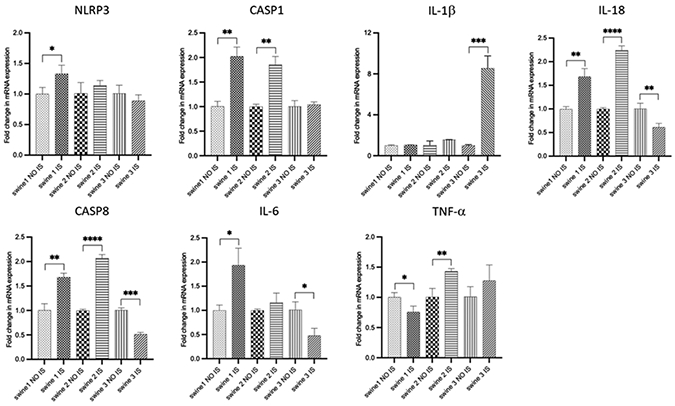
RT-PCR data for mRNA transcripts of inflammation-related genes. Comparisons between injured site (IS) vs non-injured site (NO IS) for each individual swine were performed using Student’s t test. Data are presented as mean ± SD. * p<0.05, **p<0.01, ***p<0.001, ****p<0.0001. Swine 1: untreated control; swine 2: delayed electromagnetic field (EMF) application; swine 3: immediate EMF application. NLRP3: NLR family pyrin domain containing 3; CASP1: caspase 1; IL-1β: interleukin-1 beta; IL-18: interleukin-18; CASP8: caspase 8; IL-6: interleukin-6; TNF-α: tumor necrosis factor α.

For each gene in the study, comparisons between IS *vs* contralateral NO IS were carried out for each independent animal (Figure 4). For swine 1, TBI was associated with an increase in transcriptional expression of NLRP3, CASP1, CASP8, IL-18, and IL-6, while there was no effect for IL-1β and a slight reduction for TNF-α. Swine 2 exhibited increased levels of CASP1, CASP8, IL-18, and TNF-α; no differences were observed for NLRP3, IL-1β and IL-6. For swine 3 there was a reduction in levels of CASP8, IL-18, and IL-6; there were no differences for NLRP3, CASP1, and TNF-α and there was an increase for IL-1β.

**Figure 5:**
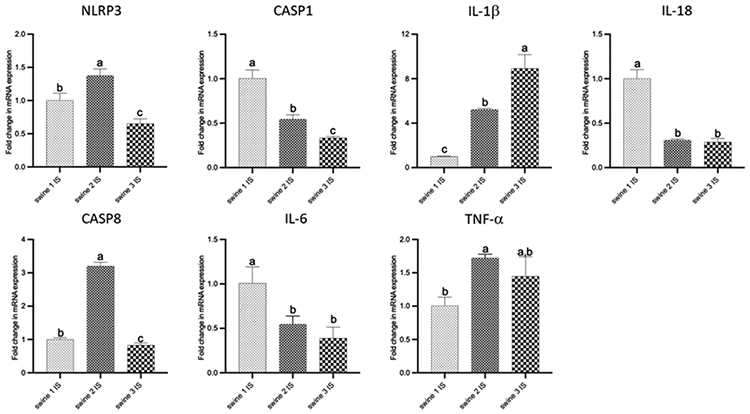
RT-PCR data for mRNA transcripts of inflammation-related genes. Comparisons among injured site (IS) from all swine were performed using an ordinary one-way ANOVA followed by Tukey's multiple comparisons test. Data are presented as mean ± SD. Different letters indicate significant differences for at least p < 0.05. Swine 1: untreated control; swine 2: delayed electromagnetic field (EMF) application; swine 3: immediate EMF application. NLRP3: NLR family pyrin domain containing 3; CASP1: caspase 1; IL-1β: interleukin-1 beta; IL-18: interleukin-18; CASP8: caspase 8; IL-6: interleukin-6; TNF-α: tumor necrosis factor α.

Comparisons among IS of all swine shown that delayed-EMF application (swine 2) was associated to a reduction in CASP1, IL-18, and IL-6 expression and an increase in NLPR3, CASP-8, IL-1β and TNF-α compared to no-EMF (swine 1). On the other hand, immediate-EMF application (swine 3) was associated with a reduction in levels of NLRP3, CASP1, CASP8, IL-18, and IL-6, while an increase in IL-1β, with no differences observed for TNF-α compared to no-EMF (swine 1). Comparison between swine 2 and swine 3 revealed differences in the mRNA transcripts of NLRP3, CASP1, CASP8, and IL-1β (Figure 5).

### Immunostaining

**Figure 6:**
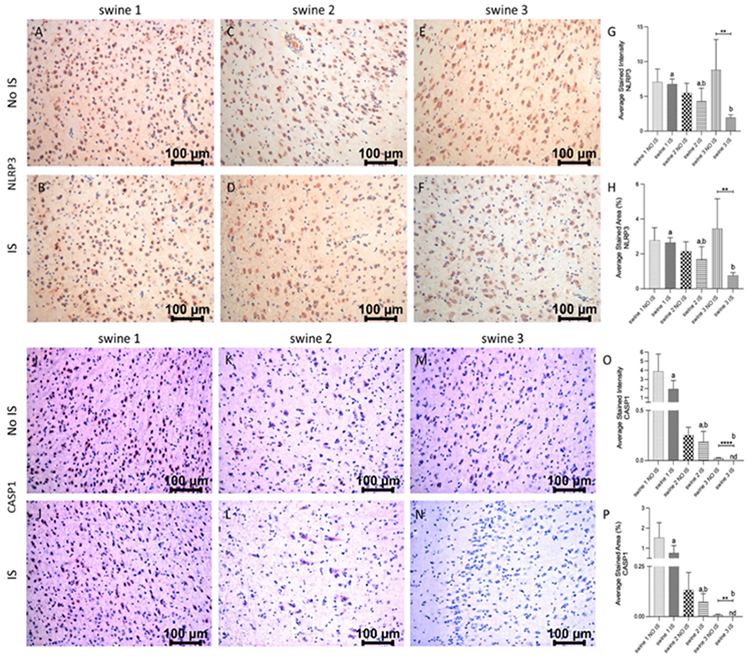
Immunohistochemistry (IHC) staining for NLR family pyrin domain containing 3 (NRLP3) and caspase (CASP)1 in non-injured (NO IS) and injured (IS) cortex tissues of Yucatan miniswine. Images are representative of all IHC studies. Comparisons between IS vs NO IS for each individual swine were performed using Student’s t test. Data are presented as mean ± SD. **p<0.01, ****p<0.0001. Comparisons among IS from all swine were performed using an ordinary one-way ANOVA followed by Tukey's multiple comparisons test. Different letters indicate significant differences for at least p < 0.05. IHC for NLRP3 (panels A-F), CASP1 (panels I-N), average stained intensity (panels G and O) and average stained percent area (panels H and P). Swine 1: untreated control; swine 2: delayed electromagnetic field (EMF) application; swine 3: immediate EMF application.

IHC of brain cortex sections revealed immunopositivity for NLRP3 and the expression levels were similar in IS and NO IS for swine 1 and swine 2. For swine 3, there was a pronounced decrease in this marker. When comparing the damaged areas of the three swine, the results revealed that immediate-EMF treatment (swine 3) was associated with a decrease in the expression of NLRP3 compared to the control animal (swine 1) (Figure 6, panels A-H). For CASP1, the highest immunoreactivity was observed in the swine 1, with no difference between IS and No IS. A positive signal was less pronounced in swine 2 and swine 3, with significantly reduced or non-detected expression in both IS and NO IS of swine 3. As for NLRP3, we detected a statistical difference between IS of swine 3 and swine 1 (Figure 6, panels I-P).

**Figure 7:**
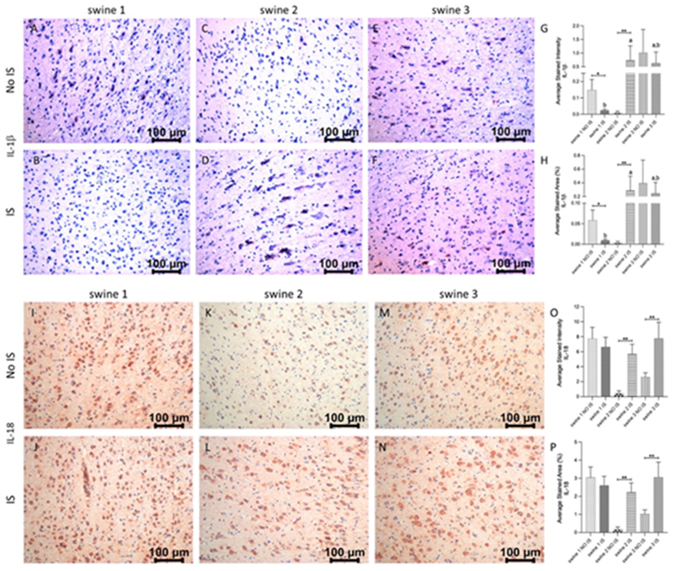
Immunohistochemistry (IHC) staining for Interleukin (IL)-1β and IL-18 in non-injured (NO IS) and injured (IS) cortex tissues of Yucatan miniswine. Images are representative of all IHC studies. Comparisons between IS vs NO IS for each animal were performed using Student’s t test. Data are presented as mean ± SD. * p<0.05, **p<0.01. Comparisons among IS from all animals were performed using an ordinary one-way ANOVA followed by Tukey's multiple comparisons test. Different letters indicate significant differences for at least p < 0.05. IHC for IL-1β (panels A-F), IL-18 (panels I-N), average stained intensity (panels G and O) and average stained percent area (panels H and P). Swine 1: untreated control; swine 2: delayed electromagnetic field (EMF) application; swine 3: immediate EMF application.

There was a significantly high immunopositivity for IL-1β in IS of swine 2 and swine 3, while tissue sections from swine 1 showed less stained intensity and area and IS was statistically different to NO IS. Immunostaining in IS of swine 2 and swine 3 was the same and statistically different from IS of swine 1 (Figure 7, panels A-H). For IL-18, immunoreactivity was similar in both brain areas of swine 1, but the positive signals in the IS from both EMF-treated animals (swine 2 and swine 3) were statistically significant compared to their respective NO IS. For this marker, immunostaining in IS of all swine was the same (Figure 7, panels I-P).

**Figure 8:**
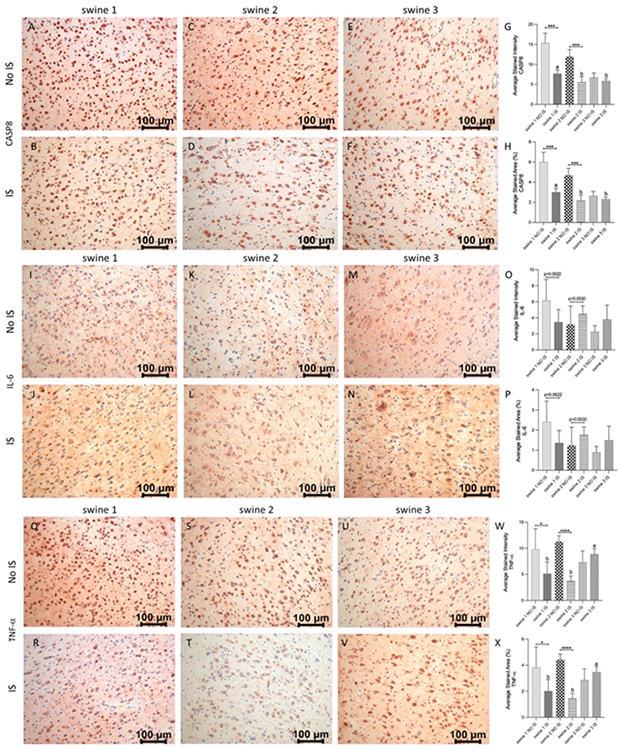
Immunohistochemistry (IHC) staining for Caspase 8 (CASP8), Interleukin (IL)-6 and Tumor Necrosis Factor α (TNF-α) in non-injured (NO IS) and injured (IS) cortex tissues of Yucatan miniswine. Images are representative of all IHC studies. Comparisons between IS vs NO IS for each individual subject were performed using Student’s t test. Data are presented as mean ± SD. * p<0.05, ***p<0.001, ****p<0.0001. Comparisons among IS from all subjects were performed using an ordinary one-way ANOVA followed by Tukey's multiple comparisons test. Different letters indicate significant differences for at least p < 0.05. IHC for CASP8 (panels A-F), IL-6 (panels I-N), TNF-α (panels Q-V), average stained intensity (panels G, O and W) and average stained percent area (panels H, P and X). Swine 1: untreated control; swine 2: delayed electromagnetic field (EMF) application; swine 3: immediate EMF application.

In the case of CASP8, there was a significant reduction of immunoreactivity in IS compared to NO IS for swine 1 and swine 2, while expression levels for swine 3 were the same on both sides of the brain. As for precedent markers, EMF was related to a significant reduction of this protein, compared to the non-EMF control area (Figure 8, panels A-H). Finally, for IL-6 there were no differences between IS and NO IS for each swine. Similarly, the expression level was the same when compared IS of the three animals (Figure 8, panels I-P). For TNF-α, there was less expression in IS compared to NO IS for swine 1 and swine 2, and similar immunostaining for both areas in swine 3. Comparing IS in all animals, there was higher intensity and stained area in swine 3 than in swine 1 and swine 2 (Figure 8, panels Q-X) for both areas in swine 3.

### Western blot

**Figure 9:**
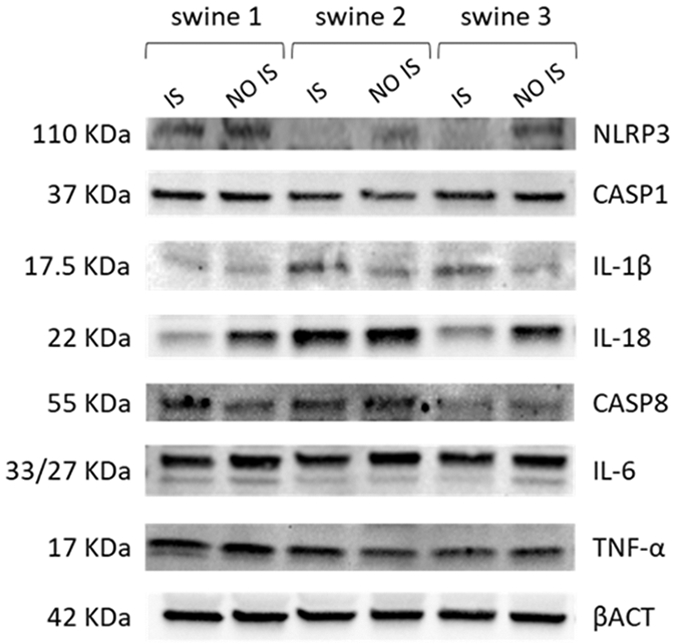
Western Blot analysis of cortex tissue from injured site (IS) and non-injured site (NO IS) of Yucatan miniswine. Swine 1: untreated control; swine 2: delayed electromagnetic field (EMF) application; swine 3: immediate EMF application. NLRP3: NLR family pyrin domain containing 3; CASP1: caspase 1; IL-1β: interleukin-1 beta; IL-18: interleukin-18; CASP8: caspase 8; IL-6: interleukin-6; TNF-α: tumor necrosis factor α; βACT: beta-actin.

Preliminary results of the Western blot study suggested that EMF could be associated with a decrease in NLRP3 protein in the IS of swine 2 and swine 3, independently of the time of application. At first glance, no changes were observed in the expression of CASP1, but a lower expression of CASP8 is evident in swine 3. The result for IL-1β corresponds to that of the transcriptional expression, as more intense bands were observed in the IS samples of swine 2 and swine 3. Likewise, this initial result corroborates the decrease in the expression of IL-18 in the IS of swine 3 and a greater expression in the same area of swine 2. On the other hand, a slight decrease in IL-6 was observed in the IS of swine 2 and swine 3, when compared with their corresponding NO IS. For TNF-α, no large variations were detected among the samples analyzed (Figure 9). As we stated before, these results are preliminary and need to be corroborated in studies that are currently planned with an increased number of swine.

### ELISA

Table 3 summarizes the values for each marker in brain cortex samples. For both EMF-treated animals (swine 2 and swine 3), markers NSE, GFAP, UCHL1, MBP, IL-1β, IL-6, and TAC showed higher concentrations in IS than in NO IS. For the control animal (swine 1), there were similar values in IS and NO IS for NSE, GFAP, UCHL1, MBP, and TAC; IL-1β was not detected in NO IS; IL-6 and TNF-α showed higher numerical values in IS than in NO IS.

**Figure 10:**
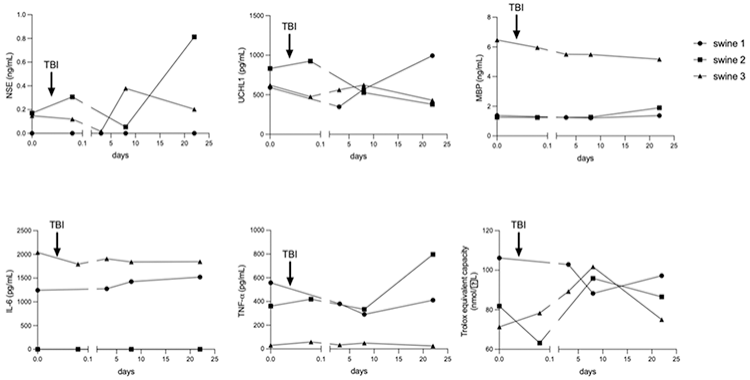
Time course variations in Traumatic Brain Injury (TBI) and inflammation markers during the development of the animal model in Yucatan miniswine, quantified by specific ELISA systems. The arrow represents the moment in which mechanical impact was applied. Swine 1: untreated control; swine 2: delayed electromagnetic field (EMF) application; swine 3: immediate EMF application. NSE: Neuron-specific enolase; UCHL1: Ubiquitin Carboxy-terminal Hydrolase; MBP: Myelin Basic Protein; IL-6: Interleukin-6; TNF-α: Tumor Necrosis Factor α; TAC: Total Antioxidant Capacity.

Results showed that each swine exhibited different concentrations for each marker in serum before TBI surgery. In the control animal (swine 1) there was a tendency concerning a numerical increase in markers such as UCHL1, TNF-α and TAC towards the final day of the study. It showed no variations in MBP levels and only a slight increase in IL-6. No NSE was detected in any of the serum samples from this animal (Figure 10, circle lines). For delayed-EMF swine (swine 2), there was a marked increase in the serum concentration of NSE and TNF-α on the final day of the study. MBP did not vary in time. UCHL1 concentration decreased, after a slight increase post-TBI. TAC showed a marked increase on day 8 but then exhibited a decreasing trend. IL-6 was not detected in any sample (Figure 10, square lines). Swine 3 exhibited final numerical values for NSE, UCHL1, TNF-α and TAC lower than swine 2 and swine 1. Since the beginning of the study, swine 3 exhibited the highest concentrations for MBP and IL-6, which didn’t markedly change in time, only a slight tendency to decrease (Figure 10, triangle lines). In general, we failed to detect GFAP and IL-1β in serum samples.

## Discussion

At the molecular level, TBI is characterized by persistent neuroinflammation, an event that occurs in the second phase of TBI and plays a fundamental role in neurodegeneration, alteration of neural and synaptic transmission, and cell death [7, 32]. During the neuroinflammatory response, peripheral immune mediators cross through the damaged blood-brain barrier and contribute to the activation of microglia, inducing the secretion of proinflammatory cytokines such as IL-1β, IL-6, and TNF-α [33]. Animal models developed in rodents have been historically used to study mechanisms of TBI impact due to its low cost and ease of handling [34]. But these models do not always reflect the complexity of the damage that takes place in the human brain and on many occasions, the positive results obtained in this type of experimental animal are not obtained in clinical trials, therefore it is necessary to address the medical problem in models closer to human anatomy. Thus, TBI models in pigs have come to overcome the anatomical gaps between rodents and humans, allowing more accurate studies of the underlying mechanisms and the evaluation of drugs and treatments with greater effectiveness.

Indeed, EMF represents a novel approach for treating complications derived from TBI. The application of this technology has shown positive effects in models of peripheral nerve injury in rodents, promoting axonal regeneration and functional recovery [35,36]. In the clinical arena, EMF has been used to treat several conditions like mirror movements, traumatic spinal cord injuries, and hemispherectomy and to stimulate the peripheral nervous system after amputations [37]. Although the results are promising, research must be carried out to demonstrate the feasibility of applying EMF in TBI. Some important concerns like optimal electromagnetic settings, molecular biology of the disease, and EMF mechanisms of action must be finely tuned before establishing this technology as a widespread therapeutic option.

After mechanical impact to the head, NLRP3 inflammasome is activated by high ATP levels, damage- and pathogen-associated molecular patterns signals, increase in intracellular calcium, cellular potassium efflux, and mitochondrial dysfunction [38,39]. NLRP3 complex is mainly located in microglia [40] and after assembly and activation, pro-caspase 1 is proteolytically processed into its active form and in turn, it converts pro-IL-1β and pro-IL-18 into IL-1β and IL-18 pro-inflammatory cytokines [9,10]. High levels of NLRP3, caspase-1, IL-1β and IL-18 have been detected in the serum of TBI patients [41,42] as well as in the injured cerebral cortex in a murine model of TBI [43]. In our research, 21 days after inducing TBI, transcriptional levels of NLRP3 complex genes were higher in IS compared to NO IS in swine 1, except for IL-1β, for which the levels were similar. At the protein level, swine 1 exhibited the highest levels of CASP1 for both areas in the brain, which could be responsible for the high expression of IL-18. IL-1β showed the lowest levels among three animals, in terms of gene and protein expression. In swine 2, the delayed application of EMF exerted a mild restorative effect by decreasing the transcriptional and protein expression of NLRP3 to the levels of the intact contralateral zone. Also in swine 2, coding RNA for CASP1 remained elevated, and this could be responsible for the higher level of IL-18 observed in IS compared to NO IS. While IL-1β transcriptional expression was the same for both, injured and no injured areas in swine 2, the significant increase of this cytokine detected in IS suggests the incidence of putative protein stabilization mechanisms.

In swine 3, the immediate application of EMF on the injured area maintained the levels of NLRP3 and CASP1 like those of the contralateral intact region, which ultimately resulted in significantly lower levels for both proteins in the IS tissue. For the IL-18 coding gene, there was a reduction in its transcriptional expression, but after 21 days protein levels remained significantly higher in IS compared to NO IS of swine 3, as observed for swine 2. Besides activation by CASP1, pro-IL-18 is also processed by several other proteases like CASP8 [44], proteinase 3 [45], mast cell chymase [46], and granzyme B [47] and it has a very wide interaction network that covers 319 molecules and 402 reactions and it is also diverse according to the cell type under study. So, it is inferred then that its post-translational regulation does not depend on a single mechanism [48]. Whether this increase is relevant in the context of TBI and EMF, should be explored in further experiments as IL-18 induces other pro-inflammatory factors like TNF-α, IL-1β, IL-6, inducible nitric oxide, and cyclooxygenase 2, among others [48]. For IL-1β, an unanticipated significant increase in transcriptional expression was obtained in IS compared to the NO IS site, although both proteins were similarly immunodetected in swine 3. This result could be contradictory when compared to the effect observed for the rest of the inflammation markers in this animal.

According to previous reports in the literature, despite being a prototypical "pro-inflammatory" cytokine, IL-1β can also provide protection to the damaged CNS. Several studies have demonstrated that IL-1β-stimulated astrocytes increase neuronal survival through the production of neurotrophic factors [49,50]. In an animal model of Parkinson’s Disease, the overexpression of IL-1β in the caudate nucleus increases tyrosine hydroxylase immunoreactivity and behavioral outcome of the animals eight weeks after lesion [51]. In a transgenic model for Alzheimer’s disease, sustained hippocampal IL-1β overexpression ameliorated β-amyloid plaques size and frequency [52,53]. IL-1β has also been shown to mediate ischemic tolerance, contributing to building a protective response [54]. Finally, IL-1β can reduce excitotoxicity neuronal cell death after the addition of ionotropic glutamate receptor agonists to primary neuronal and organotypic slice cultures [55-58]. Whether the observed increase in IL-1β transcriptional expression in our experimental model contributes to neuronal protection and/or recovery should be assessed in future studies.

Besides its major role in cell apoptosis promoted by death receptors triggering, mitochondrial apoptosis, and endoplasmic reticulum stress [59-61], CASP8 has been shown to regulate inflammation by modulating IL-1β mRNA expression, specifically by the activation of nuclear factor-kB (NF-κB) [62]. In other biological scenarios, CASP8 has also been involved in NLRP3 priming and activation, where it was directly associated with cleavage and processing of procaspase-1, IL-1β, and IL-18 processing [63-66]. We decided to study CASP8 in our model due to its contribution to neuronal pathologies like TBI [67-69], brain ischemia [70], and seizures [71]. In our study, TBI stimulated a significant increase in the transcriptional expression of this gene in the IS of swine 1 and swine 2, but this effect was not reproduced in protein expression, as a significant decrease in the immunodetection of this marker was observed for both animals. The immediate application of EMF decreased the expression of this transcript, which resulted in similar protein levels between IS and NO IS in swine 3. It has been proposed that, in the absence of CASP1, NLRP3 inflammasome employs CASP8 pro-apoptotic initiator and IL-1β-processing protease [72], so the attenuation of this marker could be crucial in the attenuation of the inflammatory process and other pathological molecular events that activate during TBI, positively contributing to neuronal recovery.

Following TBI, IL-6, and TNF-α are released by activated microglia [33] among other chemical mediators like prostaglandins, chemokines, and cell adhesion molecules [73]. TNF-α is also implicated in necrosis, which promotes cell membrane disruption and release of damage-associated molecular patterns molecules, establishing a vicious cycle of neuroinflammation and cell death [13]. It has been shown that the downregulation of NLRP3 inflammasome leads to a decrease in the production of these cytokines [74]. In our experimental conditions, mechanical injury led to an increase in transcriptional expression of IL-6 in IS of swine 1, while EMF exposure maintained or reduced its levels in IS compared to NO IS in swine 2 and swine 3. For this cytokine, no differences in immunodetection were observed, although some comparisons rendered p values very close to significance. For TNF-α, there were higher levels of transcript in IS of swine 2 compared to NO IS, but at the protein level, there was a reduction. For swine 3, no differences were found at the transcriptional level but immunodetection of protein was higher in IS. This could agree with increased IL-18 observed in this tissue, as it has been proved that this cytokine induces the production of TNF-α [75-77].

In addition to the individual comparisons between the IS and NO IS of each swine, we also compared the IS of all swine, to look for a possible effect of the moment in which the EMF is applied after TBI, on the expression of inflammatory markers in this study. The results obtained suggest that the immediate application of EMF is more effective in reducing the levels of the NLRP3 complex molecules as well as pro-inflammatory cytokines. These observations, extrapolated to the clinical scenario, speak in favor of the need to minimize the time between the occurrence of damage and the application of EMF, although more experiments should be performed to study the window of therapeutic opportunities for the treatment with different frequencies.

Serum biomarkers in TBI have been widely used as a diagnostic tool in establishing the severity of the injury, the prognosis of the illness, and the effectiveness of therapies [78]. The combination of molecular markers with more sophisticated techniques, like computed tomography and magnetic resonance, represents a powerful tool for specialists to design proper assistance protocols for each patient. In our study, besides transcriptional expression, and protein levels of inflammation-related molecules, we included quantification in tissue and serum samples of some biomarkers closely related to TBI pathogenesis. Among them, NSE is a marker for neurons and peripheral neuroendocrine cells but is also present in microglia, oligodendrocytes, and astrocytes [79]. This glycolytic enzyme converts 2-phosphoglycerate to phosphoenolpyruvate and its increase in serum has been associated with neuronal damage [80] and several neurodegenerative disorders like Parkinson’s disease, Alzheimer’s disease, Huntington’s disease, and Amyotrophic Lateral Sclerosis [81-83]. NSE has also been assessed as a TBI marker [84-87], as it appears very quickly after trauma [88], but the results associated with this marker must be interpreted with caution since NSE is also present in erythrocytes [89], therefore blood hemolysis could interfere. In our model, higher levels of tissue NSE in IS than in NO IS of swine 2 and swine 3 could be associated with a recovery of neuronal cell mass. This result should be corroborated with IHC studies. In serum, we failed to detect NSE in samples from control animal (swine 1). The final concentration in delayed-EMF treated animal (swine 2) was higher than that obtained for immediate-EMF treated animal (swine 3), and this could be associated with a worse outcome after TBI.

Another serum protein extensively employed to characterize TBI patients is UCHL1 [90]. This enzyme is also a marker for neurons [91] and is involved in ubiquitination and de-ubiquitination of proteins destined for catabolism [92, 93]. UCHL1 has been shown to rapidly increase after TBI compared to uninjured controls [94-96]. Along with GFAP, UCHL1 is one of the only two biomarkers approved by the Federal Drug Administration for patient monitoring [97]. In our TBI model, levels of brain UCHL1 in the control animal (swine 1) were the same for IS and NO IS. In EMF-treated animals (swine 2 and swine 3), concentrations in IS doubled the ones in NO IS. This increase could be beneficial for neuronal recovery as this enzyme plays an important role in the repair of axons and neurons after injury by removal of abnormal proteins by the ubiquitin–proteasome pathway [98], protects neurons from cytotoxicity [99] and also regulates synaptic function [100]. Our results showed a decrease in serum levels of UCHL1 in EMF-treated animals (swine 2 and swine 3), while the control animal (swine 1) exhibited an increase in this protein towards the end of the experiment. These findings suggest that the treatment could be associated with a reduction in this marker, which could be considered an improvement for neuronal restoration.

GFAP represents the main component of the cytoskeleton of astrocytes and is found only in glial cells of the CNS [101]. It is upregulated during astrogliosis, an activated state of astrocytes that arises after trauma or infection [102]. The presence of this protein in serum represents a specific marker of damage after head trauma [103-106]. In our study, brain GFAP levels in IS and NO IS for each swine were similar. Regarding quantification in serum, we failed to detect this protein. This could be due to the sensitivity of the ELISA system we employed, in which the detection range covers from 310 – 20,000 pg/mL, with a sensitivity of 190 pg/mL. According to previous results, serum GFAP in TBI models in minipigs could vary from 0 to 200 pg/mL, with an average value near 100 pg/mL [107, 108]. Taking this into account, a more sensitive system should be employed in future studies.

MBP is the second most abundant protein in the CNS, and it is specific to the myelin sheets. This protein constitutes a biomarker for oligodendrocytes which binds the cytoskeleton to the cell membrane and mediates extracellular signals to the cytoskeleton [109]. Circulating levels of MBP increase after brain damage [110,111] or demyelinating diseases [112]. In our model, we detected higher levels of brain MBP in IS compared to NO IS for EMF-treated animals (swine 2 and swine 3). This result could be indicative of an axonal system recovery driven by a higher presence of oligodendrocytes, but this should be confirmed in histological studies. Levels of MBP in serum did not vary over time, in agreement with previous reports [113], suggesting that this protein might not be used as a TBI biomarker, but it could be useful as a mature oligodendrocyte indicator.

TBI is characterized by high levels of circulating pro-inflammatory cytokines such as IL-1β, IL-6, and TNF-α [33, 114]. These cytokines are closely related, as TNF-α induces the expression of IL-1β and IL-6 and, in turn, IL-1β induces IL-6 and TNF-α, triggering a pro-inflammatory loop [115]. Accordingly, they can be used as biomarkers to study the pathological entity and to evaluate the effectiveness of different therapies [42, 116-118]. In our model, the highest concentration of IL-1β corresponded to IS of swine 3, and the lowest values for swine 1, coinciding with qPCR and IHC results. Serum levels of IL-1β could not be detected with the ELISA system we employed. IL-6 protein showed higher levels in IS of all animals compared to corresponding NO IS. This result differed from that obtained in the IHC study, where immunoreactivity was similar in both areas of each animal. In serum, we did not detect IL-6 in samples from swine 2, and for swine 1 and swine 3, initial and final values were similar, only with slight variations among different sampling times. TNF-α values exhibited a high increase in IS compared to NO IS in swine 1 and swine 3 and a decrease in swine 2. This result was also discordant to IHC observations. In serum, opposite to tissue values, swine 3 showed the lowest concentrations, with no variations in time. Only swine 2 exhibited a rise in TNF-α at the end of the study. According to the wide variability in serum levels and in the fluctuation pattern over time, at least in this study, these pro-inflammatory cytokines should not be considered as exclusive biomarkers of damage or recovery. To accurately establish its usefulness in this sense, it would be necessary to increase the number of animals in the study.

Among the molecular events that take place during the second phase of damage after trauma, oxidative stress stands out, closely related to mitochondrial dysfunction and neuroinflammation [119,120]. The excessive influx of Ca^2+^ contributes to mitochondrial failure and the subsequent overproduction of ROS and their derivatives [121]. In physiological conditions, cells control oxidative stress by several mechanisms including enzymatic and non-enzymatic elements such as catalase, superoxide dismutase, glutathione peroxidase, uric acid, glutathione, and ascorbic acid, among others [122]. However, during pathological circumstances like TBI, there is an imbalance in favor of the production of free radicals, and this results in neuronal degeneration and loss of physiological functions [123]. TAC of a given tissue provides relevant biological information about how prepared it is to respond to pathological increased oxidative stress. This measurement includes all categories of antioxidant species: enzymes, small molecules, and proteins. In our model, EMF application was associated with an increase in this parameter in IS compared to NO IS in swine 2 and swine 3, which represents an increase of local mechanisms to diminish oxidative stress. Regarding TAC in serum, in swine 2 and swine 3, it is noteworthy the increase observed during the first eight days of the study could be considered an effect stimulated by the EMF treatment in response to TBI-elicited damage. The reduction in TAC towards the end of the study could be indicative of a reduction in circulating levels of oxidative stress markers. This effect should be corroborated in future studies by measuring total oxidative capacity and levels of oxidative stress related molecules.

## Conclusions

The present pilot study reveals promising effects of the EMF application to reverse the deleterious consequences of neuroinflammation as one of the TBI pathological elements. Furthermore, the results suggest that the early application of the treatment could provide better protection to patients, as a more generalized effect on the reduction of inflammatory markers was observed at the transcriptional and translational levels. Although encouraging evidence, further studies should be carried out to confirm these results by expanding the number of experimental animals, as well as to verify whether the treatment equally attenuates other pathological events that characterize TBI such as oxidative stress, excitotoxicity, apoptosis, mitochondrial damage, and neuronal degeneration.

### Limitations of the study

The major limitation of this study is the low sample size of swine in each experimental group. This restrains the possibility of reaching much more supported conclusions regarding the effectiveness of the treatment, although it does provide useful information and serves to pave the way for further studies increasing the number of animals to validate the initial findings.

## Figures and Tables

**Figure 3a:**
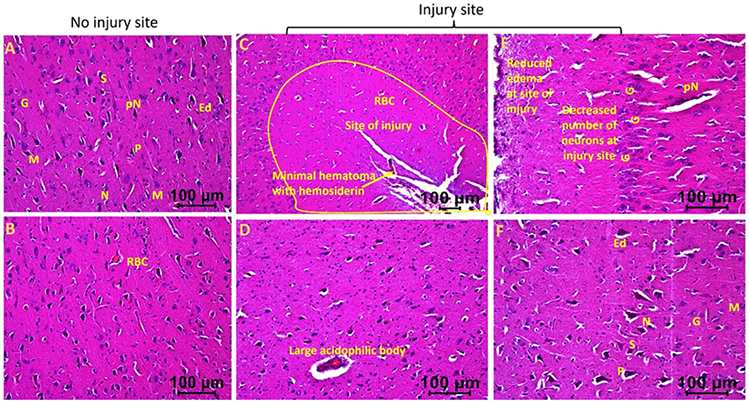
Hematoxylin and eosin staining in swine with TBI with EMF application immediately after injury (swine 3). dN- Deformed Neuron, dS- Deformed Stellate cell, dM- deformed Microglia, dP- Deformed Pyramidal cells, pN- Neurons with pyknosis, gN- Ghost Neuron, Ed- Edema around neuron and stellate cells, rER- Regenerating Endoplasmic Reticulum, M- Microglia, G- Granular cell. These are represented images of all images scanned in this swine. The images were scanned at 100μm.

**Figure 3b:**
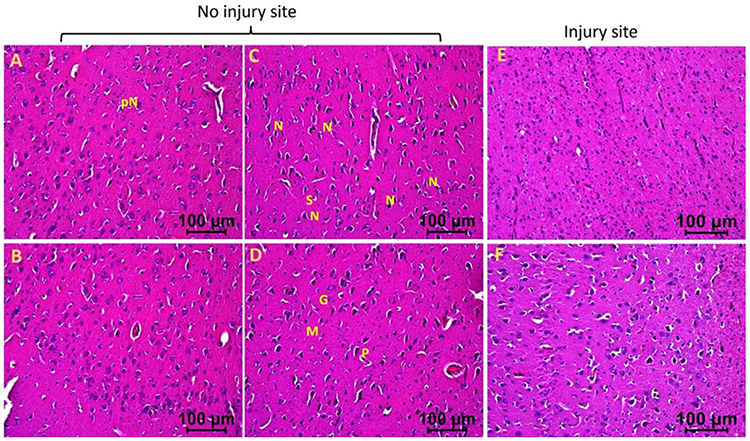
Hematoxylin and eosin staining in swine with TBI with EMF application immediately after injury (swine 3). dN- Deformed Neuron, dS- Deformed Stellate cell, dM- deformed Microglia, dP- Deformed Pyramidal cells, pN- Neurons with pyknosis, gN- Ghost Neuron, Ed- Edema around neuron and stellate cells, rER- Regenerating Endoplasmic Reticulum, M- Microglia, G- Granular cell. These are represented images of all images scanned in this swine. The images were scanned at 100μm.

**Table 1:** Sequences of forward and reverse oligonucleotides used in this study for gene of interest amplification by RT-qPCR. 18S gene was used as a housekeeping gene to normalize results. NLRP3: NLR family pyrin domain containing 3; CASP1: caspase 1; IL-1β: interleukin-1 beta; IL-18: interleukin-18; CASP8: caspase 8; IL-6: interleukin-6; TNF-α: tumor necrosis factor α.

Gene name	Forward	Reverse
NLRP3	5’-CGAGACGTGACAGTTCTTCTT-3’	5’-GGACGTTCTCTCCTGGTTTAC-3’
CASP1	5’-GGGTTACAGTGTGGATGTTAGAG-3’	5’-CATGAGACATGAGCACCAGAA-3’
IL-1β	5’-TGCATGAGCTTTGTGCAAGGAG-3’	5’-AGGGTGGGCGTGTTATCTTTCA-3’
IL-18	5’-TACGAAATCTGAACGACCAAGT-3’	5’-ATACGGTCTGAGGTGCATTATC-3’
CASP8	5’-TATATCCCAGACGAGGCGGACT-3’	5’-TTCTTTCAGGCTCTGGCACAGT-3’
IL-6	5’-CTGATCCAGACCCTGAGGCAAA-3’	5’-ACTCGTTCTGTGACTGCAGCTT-3’
TNF-α	5’-TTCCTCACTCACACCATCAGCC-3’	5’-GGTAGATGGGTTCGTACCAGGG-3’
18S	5’-CCCACGGAATCGAGAAAGAG-3’	5’-TTGACGGAAGGGCACCA-3’

**Table 2:** Primary and secondary antibodies dilution factors for Immunohistochemistry and Western blot. NLRP3: NLR family pyrin domain containing 3; CASP1: caspase 1; IL-1β: interleukin-1 beta; IL-18: interleukin-18; CASP8: caspase 8; IL-6: interleukin-6; TNF-α: tumor necrosis factor α; IHC: immunohistochemistry; WB: Western blot.

Antibody	Catalog	Dilution in IHC	Dilution in WB
**Primary antibodies**			
NLRP3	AP32694PU-N	1:50	0.3 μg/mL
CASP1	PA5-119001	1:100	1:1000
IL-1β	MBS2025860	1:100	1:1000
IL-18	MBS2026569	1:100	1:1000
CASP8	ABIN724205	1:100	1:2000
IL-6	MBS2005254	1:100	1:1000
TNF-α	MBS820357	1:100	1:1000
ACTB	ab8226	-	1:1000
**Secondary antibodies**			
Anti-mouse	BP-2000-50	Ready-to-use	-
Anti-rabbit	BP-9100-50	Ready-to-use	-
Anti-goat	BP-9500-50	Ready-to-use	-
Anti-mouse	NB7544	-	1:3000
Anti-rabbit	A16023	-	1:2000
Anti-goat	STAR206P	-	1:5000

**Table 3:** Levels of TBI and inflammation markers in brain cortex samples collected at day 21 during terminal surgery. IS: Injury site; NO IS: No injury site; NSE: Neuron Specific Enolase; GFAP: Glial Fibrillary Acidic Protein; UCHL1: Ubiquitin Carboxy-terminal Hydrolase; MBP: Myelin Basic Protein; IL-1β: Interleukin-1 beta; IL-6: Interleukin-6; TNF-α: Tumor Necrosis Factor α; TAC: Total Antioxidant Capacity. Swine 1: untreated control. swine 2: delayed electromagnetic field (EMF) application. swine 3: immediate EMF application. nd: not detected.

	Swine 1 NO IS	Swine 1 IS	Swine 2 NO IS	Swine 2 IS	Swine 3 NO IS	Swine 3 IS
NSE (ng/mg)	0.77	0.72	0.73	1.17	0.76	1.19
GFAP (ng/mg)	2.37	2.01	1.86	2.22	1.97	2.73
UCHL1 (pg/mg)	21.92	24.82	22.95	48.87	24.11	47.97
MBP (ng/mg)	0.74	1.18	0.75	1.3	0.77	1.66
IL-1b (pg/mg)	4.55	nd	5.68	11.48	6.57	40.56
IL-6 (pg/mg)	92.83	120.26	59.98	156.06	92.61	124.95
TNF-a (pg/mg)	163.9	336.83	252.36	48.04	159.89	420.6
TAC (nmol/mg)	4.01	5.23	4.21	7.11	4.5	6.32
